# HPLC-MS/MS Analysis of *Aconiti Lateralis Radix Praeparata* and Its Combination with Red Ginseng Effect on Rat CYP450 Activities Using the Cocktail Approach

**DOI:** 10.1155/2020/8603934

**Published:** 2020-03-09

**Authors:** Wenjuan Ma, Wei Wang, Xuhua Huang, Guangzhe Yao, Qi Jia, Jiayuan Shen, Huizi Ouyang, Yanxu Chang, Jun He

**Affiliations:** Tianjin State Key Laboratory of Modern Chinese Medicine, Tianjin University of Traditional Chinese Medicine, Tianjin 301617, China

## Abstract

Red ginseng is often combined with *Aconiti Lateralis Radix Praeparata* to reduce alkaloids-related toxicity of the latter. Such herb-pairing also results in better therapeutic effect in heart failure, as compared to the singular use of either herb. The purpose of this study was to investigate the effect of *Aconiti Lateralis Radix Praeparata* and its combination with red ginseng on the activities of CYP450 enzymes in rats. A sensitive and reliable HPLC-MS/MS method was established and validated for the simultaneous determination of eight probe drugs, phenacetin (CYP1A2), tolbutamide (CYP2C9), omeprazole (CYP2C19), dextromethorphan (CYP2D6), dapsone (CYP3A4), 7-hydroxycoumarin (CYP2A6), bupropion (CYP2B6), and amodiaquine (CYP2C8), in rat plasma using diazepam as internal standard (IS). The chromatographic separation was performed on a Waters XBridge™ C18 column (2.1 mm × 100 mm, 3.5 *μ*m) using a gradient elution with the mobile phase consisting of acetonitrile and water (containing 0.1% formic acid) at a flow rate of 0.3 mL/min. The method was successfully applied in evaluating the effect of *Aconiti Lateralis Radix Praeparata* and red ginseng on the activities of CYP450 enzymes. The pharmacokinetic results of the eight probe drugs suggested that *Aconiti Lateralis Radix Praeparata* may inhibit the activity of CYP2A6, CYP2C19, CYP2B6, CYP1A2, CYP3A4, and CYP2C9 enzymes in rats. Comparison between the two groups, *Aconiti Lateralis Radix Praeparata* combined with red ginseng and *Aconiti Lateralis Radix Praeparata*, indicated that red ginseng may inhibit the activity of CYP2D6 and CYP2B6 enzymes while inducing the activity of CYP1A2, CYP3A4, and CYP2C9 enzymes.

## 1. Introduction

*Aconiti Lateralis Radix Praeparata* (“Fuzi” in Chinese), recorded in Chinese Pharmacopoeia (2015 edition), is the lateral root of *Aconitum carmichaelii* Debx., belonging to family Ranunculaceae. The major chemical components of *Aconiti Lateralis Radix Praeparata* are alkaloids including diester diterpenoid alkaloids, monoester diterpenoid alkaloids, and lipid alkaloids. *Aconiti Lateralis Radix Praeparata* has multiple pharmacological properties, including antiarrhythmia, anti-inflammatory, antitumor, and antiaging properties, but it has very narrow therapeutic range due to its severe toxicity [1–4]. Clinically, *Aconiti Lateralis Radix Praeparata* is commonly prescribed with red ginseng (“Hongshen” in Chinese, derived from the steamed root and rhizome of *Panax ginseng* C. A. Mey.) for treating heart failure [5]. Previous studies have found that the combination of red ginseng and *Aconiti Lateralis Radix Praeparata* is able to reduce the contents of diester diterpenoid alkaloids (hypaconitine and deoxyaconitine) and increase the contents of monoester diterpenoid alkaloids (benzoylmesaconine and benzoylhypaconine) in *Aconiti Lateralis Radix Praeparata*, thus strengthening the therapeutic effect of *Aconiti Lateralis Radix Praeparata* while decreasing its toxicity [6, 7]. However, the mechanism of action of *Aconiti Lateralis Radix Praeparata* combined with red ginseng is still unclear in vivo.

CYP450 family is the most important drug-metabolizing system, playing a vital role in the metabolism of endogenous and exogenous compounds [8, 9]. It is responsible for the metabolism of over 70–80% of the rate-limiting phase I metabolism of drugs [10]. More than 90% of clinical drugs are metabolized by the CYP450 enzymes, including CYP1A2, CYP2C9, CYP2C19, CYP2D6, CYP3A4, CYP2A6, CYP2B6, and CYP2C8 [11]. Clinically, induction or inhibition of the CYP450 enzyme activities has been regarded as the foremost contributor to assess drug-drug interactions (DDIs) [12]. Evaluation of the effect of drugs, especially the combination of traditional Chinese medicine, on CYP450 enzyme activities is increasingly important, since it can explain the mechanism of action of DDIs or predict the potential side effects [13, 14]. Recent studies identified that CYP450 enzymes such as CYP2D6 and CYP3A4 were involved in the metabolism of *Aconiti Lateralis Radix Praeparata*-red ginseng herbal formulation. Compounds such as Ginsenosides Rc, Rf, and Rb2 induced CYP3A4 metabolic enzymes [15, 16], while diester alkaloids of *Aconiti Lateralis Radix Praeparata* were identified as substrates of CYP3A4 metabolic enzymes [17]. However, several Ginsenoside components or in vitro studies have not been able to fully elucidate the effects of *Aconiti Lateralis Radix Praeparata* and its combination with red ginseng on CYP450 enzymes.

Cocktail approach has become one of the basic analytical tools to assess the impact of DDIs on CYP450 enzymes in vivo. This method not only provides information of multiple CYP450 enzymes activities in a single experiment but also minimizes the intersubject and intrasubject variability [18–20]. Compounds specifically catalyzed by each CYP450 enzyme, regarded as probe drugs, have been widely used to evaluate the activity of CYP450 enzymes in this method [18, 21]. CYP450 enzymes inhibition or induction by one drug could lead to the increase or reduction in plasma concentrations of another drug, thereby enhancing or attenuating the therapeutic effect [22]. In this study, a rapid and sensitive LC-MS/MS method was established for the quantitation of specific probe drugs in rat plasma to evaluate the influence of *Aconiti Lateralis Radix Praeparata* and its combination with red ginseng on CYP450 enzymes. The results would be significant in explaining the mechanism of toxic-reduction and therapeutic-enhancing effects of *Aconiti Lateralis Radix Praeparata* and red ginseng, which would provide a theoretical basis for rational usage of red ginseng and *Aconiti Lateralis Radix Praeparata* in clinical.

## 2. Experimental

### 2.1. Chemicals, Reagents, and Materials

Acetonitrile (chromatographic purity) and methanol (chromatographic purity) were purchased from Merck Co., Ltd. Formic acid (chromatographic purity) was obtained from ROE Co., Ltd. Ultrapure water was prepared with a Milli-Q water purification system (Millipore, Milford, MA, USA). Omeprazole, dextromethorphan, bupropion, 7-hydroxycoumarin, and amodiaquine were purchased from the China National Institutes for Food and Drug Control. Phenacetin, tolbutamide, and diazepam were obtained from Tianjin Vientiane Hengyuan Technology Co., Ltd. Dapsone was purchased from J&K Scientific Ltd. The extracts of red ginseng and *Aconiti Lateralis Radix Praeparata* were made in the laboratory, both of which were extracted with 70% ethanol. The extraction rates of red ginseng and *Aconiti Lateralis Radix Praeparata* were 43.36% and 15.38%, respectively.

### 2.2. Chromatographic and Mass Spectrometry Conditions

The HPLC-MS/MS system consists of an Agilent 1200 high-performance liquid chromatography coupled with an Agilent 6430 series triple quadrupole mass spectrometer with an electrospray ionization (ESI) source. The chromatographic separation was performed on a Waters XBridge™ C18 column (2.1 × 100 mm, 3.5 *μ*m) maintained at 30°C. Mobile phases consisting of 0.1% formic acid in water (A) and acetonitrile (B) were used in the following gradient elution method: 0–2 min, 10%–30% B; 2–7 min, 30%–80% B; and 7–10 min, 80%–80% B. The flow rate was 0.3 mL/min, and the injection volume was 5 *μ*L. All data were analyzed by Mass Hunter workstation software (Agilent Technologies, USA).

The mass spectrometer was carried out in positive ionization multiple reaction monitoring (MRM) mode. The optimum MS values were maintained as follows: nebulizing gas pressure, 25 psi; drying gas (N_2_) flow rate, 11 L/min; and capillary temperature, 350°C. The precursor-product ion pairs used for the MRM detection and MS parameters are shown in Table 1.

### 2.3. Preparation of *Aconiti Lateralis Radix Praeparata* and Red Ginseng Solution

The dosage of *Aconiti Lateralis Radix Praeparata* extract was 0.415 g/kg. According to the raw herb combination ratios of *Aconiti Lateralis Radix Praeparata* and red ginseng (1 : 2), the dosage of red ginseng extract was 2.34 g/kg. Appropriate amounts of *Aconiti Lateralis Radix Praeparata* and red ginseng were weighed, prepared with 0.5% CMC-Na suspension solution, and stored at 4°C.

### 2.4. Preparation of Calibration Standards and Quality Control Samples

In order to make the stock solutions (1 mg/mL), phenacetin, tolbutamide, omeprazole, dextromethorphan, dapsone, 7-hydroxycoumarin, bupropion, amodiaquine, and diazepam (internal standard) were separately weighed and dissolved in methanol. The working solutions of the eight probe drugs were prepared by mixing and diluting the primary stock solutions with methanol to obtain concentrations of 50–25000 ng/mL for 7-hydroxycoumarin; 10–5000 ng/mL for phenacetin, bupropion, and amodiaquine; 5–2500 ng/mL for dextromethorphan and dapsone; 30–15000 ng/mL for tolbutamide; and 20–10000 ng/mL for omeprazole. The working solution of diazepam (IS) was prepared at a concentration of 1000 ng/mL in methanol.

The calibration standard solutions were prepared by adding 20 *μ*L of working solutions and 20 *μ*L of IS solutions to 100 *μ*L blank plasma to give nominal concentration range of 10–5000 (10, 25, 50, 100, 250, 500, 1000, 2500, and 5000) ng/mL for 7-hydroxycoumarin; 2–1000 (2, 5, 10, 20, 50, 100, 200, 500, and 1000) ng/mL for phenacetin, bupropion, and amodiaquine; 1–500 (1, 2.5, 5, 10, 25, 50, 100, 250, and 500) ng/mL for dextromethorphan and dapsone; 6–3000 (6, 15, 30, 60, 150, 300, 600, 1500, and 3000) ng/mL for tolbutamide; and 4–2000 (4, 10, 20, 40, 100, 200, 400, 1000, and 2000) ng/mL for omeprazole.

Quality control (QC) samples at three concentrations (low, medium, and high concentration) were made up of appropriate mixed standard solutions with blank plasma and IS solutions as calibration standard solutions to meet the required concentrations. All the solutions were stored at 4°C.

### 2.5. Sample Preparation

100 *μ*L plasma sample, 20 *μ*L IS (diazepam, 1000 ng/mL), and 20 *μ*L methanol were added to a centrifuge tube and mixed by vortexing. Liquid-liquid extraction was performed by adding 1 mL ethyl acetate to the centrifuge tube. The mixture was then vortexed for 3 min and centrifuged at 14,000 rpm for 10 min. The supernatant was transferred into a new tube and evaporated to dryness under a nitrogen stream. The residue was reconstituted with 100 *μ*L methanol and vortexed for 3 min, followed by centrifugation at 14,000 rpm for 10 min. 5 *μ*L supernatant was injected into the LC-MS/MS system for analysis.

### 2.6. Method Validation

The method was validated with respect to specificity, linearity, accuracy, precision, extraction recovery, matrix effect, and stability according to bioanalytical method validation.

#### 2.6.1. Specificity

The specificity of the method was investigated by comparing chromatograms of blank plasma samples from six different rats, blank plasma spiked with the eight probe drugs and IS, and a rat plasma sample after the tail vein administration of cocktail probes.

#### 2.6.2. Linearity and LLOQ

Calibration curves were achieved using a series of calibration standard samples and constructed by plotting the peak area ratio analyte to IS (*y*) versus the concentration of analyte in spiked plasma samples (*x*), using 1/*x*^2^ as a weighing factor. The lower limit of quantification (LLOQ) was determined at a signal-to-noise ratio of about 10 by analyzing the standard plasma samples.

#### 2.6.3. Precision and Accuracy

The precision and accuracy were evaluated by determination of QC samples in six replicates at low, medium, and high concentration levels. The precision and accuracy were analyzed for 3 consecutive days with the standard calibration curve. Intra- and interday precisions were expressed as the relative standard deviation (RSD), while the accuracy was determined by the relative error (RE).

#### 2.6.4. Extraction Recovery and Matrix Effect

The extraction recovery and matrix effect of eight analytes were calculated at three concentration levels, each in six replicates. The extraction recovery was calculated using the ratio of peak area of the extracted samples and that of postextraction spiked samples. The matrix effect was obtained by comparing the peak areas of the postextraction spiked samples versus standard solutions.

#### 2.6.5. Stability

The stability of eight probe drugs was analyzed in six replicates at three concentration levels. The QC samples were prepared for 4 h at room temperature, three freeze-thaw cycles, 12 h in autosampler, and 14 days at −70°C.

### 2.7. Pharmacokinetic Study

Male Sprague-Dawley rats (190–230 g) were obtained from Beijing HFK Experimental Animal Technology Co., Ltd. The rats were fed with standard diet and water in a stable environment, with temperatures between 23 and 26°C and relative humidity of 40–60%. After a week of acclimation, the animals were fasted 12 h before the PK experiment and allowed free access to water during the experiment. 18 rats were randomly divided into three groups. The rats were given 10 mL/kg cocktail solution (2 mg/kg of 7-hydroxycoumarin; 0.2 mg/kg of tolbutamide; 1 mg/kg of omeprazole, dextromethorphan, and bupropion; 0.5 mg/kg of dapsone and phenacetin; and 3 mg/kg of amodiaquine) through the tail vein in group A. The rats were administered 0.415 g/kg *Aconiti Lateralis Radix Praeparata* extract by oral administration for 7 consecutive days and then dosed 10 mL/kg cocktail solution through the tail vein on the eighth day in group B. The rats of group C were administered 0.415 g/kg *Aconiti Lateralis Radix Praeparata* extract and 2.34 g/kg red ginseng extract by oral administration for 7 consecutive days and dosed 10 mL/kg cocktail solution through the tail vein on the eighth day. Blood samples of each rat were collected from the fossa orbitalis vein at time points 0.03, 0.08, 0.17, 0.25, 0.33, 0.50, 0.75, 1, 2, 4, 6, 8, 10, 12, and 24 h after tail vein administration. The blood samples (200 *μ*L) were transferred into heparinized microcentrifuge tubes and centrifuged at 6000 rpm for 10 min. All plasma samples were stored at –70°C. The animal studies described in this paper were approved and conducted in accordance with the guidelines of Laboratory Animal Ethics Committee of Tianjin University of Traditional Chinese Medicine (TCM-LAEC20180057).

### 2.8. Statistical Analysis

Data were presented as mean ± SD. Pharmacokinetic parameters were calculated by the computer program “Drug and Statistics 1.0” (DAS 1.0; Medical College of Wannan, China) and analyzed with one-way-ANOVA by SPSS 17.0 statistical software.

## 3. Results and Discussion

### 3.1. Optimization of HPLC-MS/MS Conditions

Acetonitrile was selected as the mobile phase as it has strong elution ability. The responses of eight probe drugs and IS were improved after adding 0.1% formic acid to water. The analytes have good retention time, peak symmetry, and appropriate ionization in the mobile phase with gradient elution.

The eight probe drugs were monitored in positive and negative ion modes, and it was discovered that ions in positive mode had more stable and stronger MS signal than in negative mode. The transitions *m*/*z* 346.2/136.1 for omeprazole, 249.1/92.1 for dapsone, 272.2/147.1 for dextromethorphan, 180.1/110.1 for phenacetin, 271.1/91.0 for tolbutamide, 240.1/184.1 for bupropion, 356.2/283.1 for amodiaquine, 163.0/107.1 for 7-hydroxycoumarin, and 285.0/193.0 for diazepam (IS) were chosen for the quantification studies.

### 3.2. Method Validation

#### 3.2.1. Specificity

**Figure 1:**
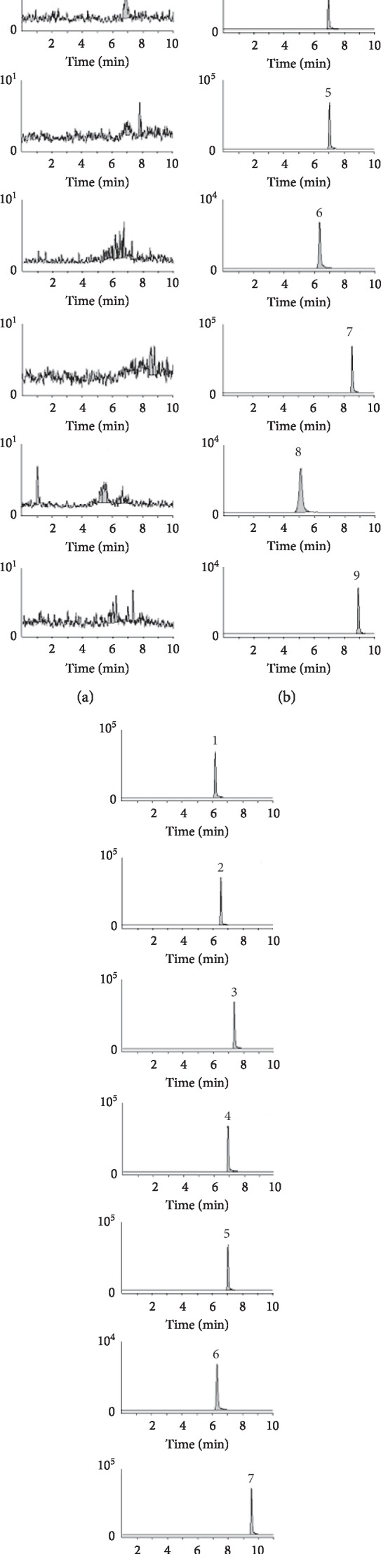
Representative MRM chromatograms of probe drugs and IS in rat plasma samples. (a) Blank plasma sample; (b) plasma sample from a rat after an intravenous administration; (c) blank plasma sample spiked with probe drugs and IS. 1. 7-Hydroxycoumarin; 2. omeprazole; 3. dextromethorphan; 4. bupropion; 5. phenacetin; 6. dapsone; 7. tolbutamide; 8. amodiaquine; and 9. diazepam (IS).

The representative chromatograms of a blank plasma (A), a rat plasma sample after the tail vein administration of cocktail probes at 10 mL/kg (B), and blank plasma sample spiked with probe drugs and IS (C) are shown in Figure 1. As shown in Figure 1, no significant interfering peaks from endogenous substance were observed at the corresponding analytes retention time.

#### 3.2.2. Linearity and LLOQ

The curves were linear (*r* > 0.991) in the calibration ranges of each probe drug in rat plasma. The regression equations, linear ranges, correlation coefficients, and LLOQ are displayed in Table 2. The results indicated that the method was linear and sensitive.

#### 3.2.3. Precision and Accuracy

As shown in Table 3, the intra- and interday precisions at three different concentration levels of eight probe drugs were 13.0% or less, while the accuracy ranged from −14.0% to 14.4%. The results of the precision and accuracy met the criteria for the analysis of biological samples.

#### 3.2.4. Extraction Recovery and Matrix Effect

The mean extraction recoveries in rat plasma ranged from 85.0% to 109.4% for the eight probe drugs at three concentration levels. The mean matrix effects of the eight analytes were between 81.5% and 105.6%. The extraction recovery and matrix effect data are summarized in Table 4.

#### 3.2.5. Stability

The results of stability are displayed in Table 5. Eight probe drugs were stable with RSD range of 0.4%–14.3% under various storage conditions.

### 3.3. Effects of *Aconiti Lateralis Radix Praeparata* and Its Combination with Red Ginseng on CYP450 Activities in Rats

**Figure 2:**
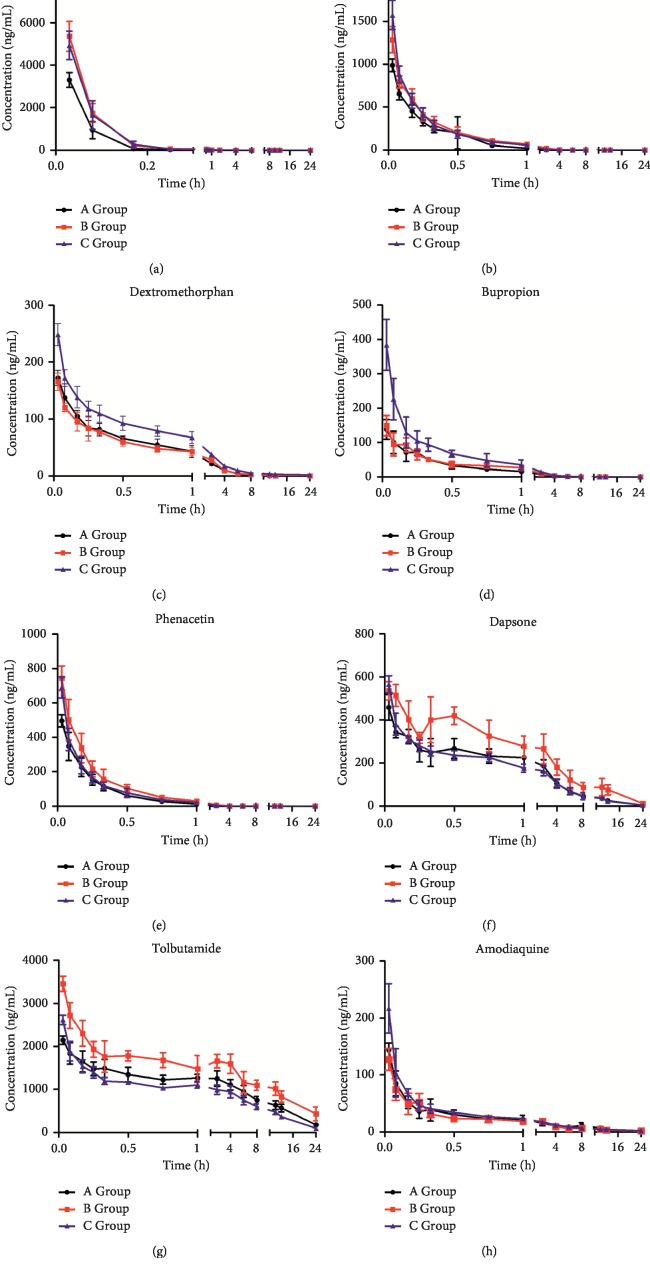
Mean plasma concentration-time curves of probe drugs in different groups (mean ± SD, *n* = 6). Group A received 10 mL/kg cocktail solution through the tail vein; group B received 0.415 g/kg *Aconiti Lateralis Radix Praeparata* extract by oral administration for 7 consecutive days and dosed 10 mL/kg cocktail solution through the tail vein on the eighth day; group C received 0.415 g/kg *Aconiti Lateralis Radix Praeparata* extract and 2.34 g/kg red ginseng extract by oral administration for 7 consecutive days and dosed 10 mL/kg cocktail solution through the tail vein on the eighth day. (a) 7-Hydroxycoumarin. (b) Omeprazole. (c) Dextromethorphan. (d) Bupropion. (e) Phenacetin. (f) Dapsone. (g) Tolbutamide. (h) Amodiaquine.

The method was applied to pharmacokinetic study of eight probe drugs in rats. The mean plasma concentration-time curves of the eight cocktail probe drugs are shown in Figure 2, and the main pharmacokinetic parameters of eight probe drugs are summarized in Table 6.

#### 3.3.1. Effects of Aconiti Lateralis Radix Praeparata on Eight Probe Drugs in Rats

Pharmacokinetic profiles of eight probe drugs after *Aconiti Lateralis Radix Praeparata* treatment were used to estimate the activity of CYP450 enzymes in rats. As compared to group A, C_max_, AUC_(0-24)_, and AUC_(0-∞)_ of 7-hydroxycoumarin, omeprazole, bupropion, phenacetin, dapsone, and tolbutamide in group B were increased, and MRT_(0-24)_ of phenacetin, dapsone, and tolbutamide were significantly added (*P* < 0.05 or *P* < 0.01). The results suggested that *Aconiti Lateralis Radix Praeparata* may inhibit the activity of CYP2A6, CYP2C19, CYP2B6, CYP1A2, CYP3A4, and CYP2C9 enzymes in rats. The pharmacokinetic parameters of dextromethorphan and amodiaquine in group B had no significant differences when compared to group A, implying that *Aconiti Lateralis Radix Praeparata* showed little alteration on CYP2D6 and CYP2C8 activity in vivo.

#### 3.3.2. Effects of Aconiti Lateralis Radix Praeparata Combination with Red Ginseng on Eight Probe Drugs in Rats

Alteration in CYP450 enzymes activity caused by red ginseng was estimated by comparing the pharmacokinetic results of eight probe drugs in rats by different treatment methods. Compared with group B, C_max_, AUC_(0-24)_, and AUC_(0-∞)_ of dextromethorphan and bupropion in group C were significantly increased (*P* < 0.01), while the half-life (*t*_1/2_) of dextromethorphan and bupropion was markedly reduced (*P* < 0.01). The results showed that metabolism of dextromethorphan and bupropion in group C was slowed. But C_max_, AUC_(0-24)_, and AUC_(0-∞)_ of phenacetin, dapsone, and tolbutamide were significantly decreased (*P* < 0.05 or *P* < 0.01), indicating that red ginseng may enhance the metabolism of phenacetin, dapsone, and tolbutamide. Thus, these pharmacokinetic parameters suggested that red ginseng may inhibit the activity of CYP2D6 and CYP2B6 enzymes but induce the activity of CYP1A2, CYP3A4, and CYP2C9 enzymes in rats.

**Figure 3:**
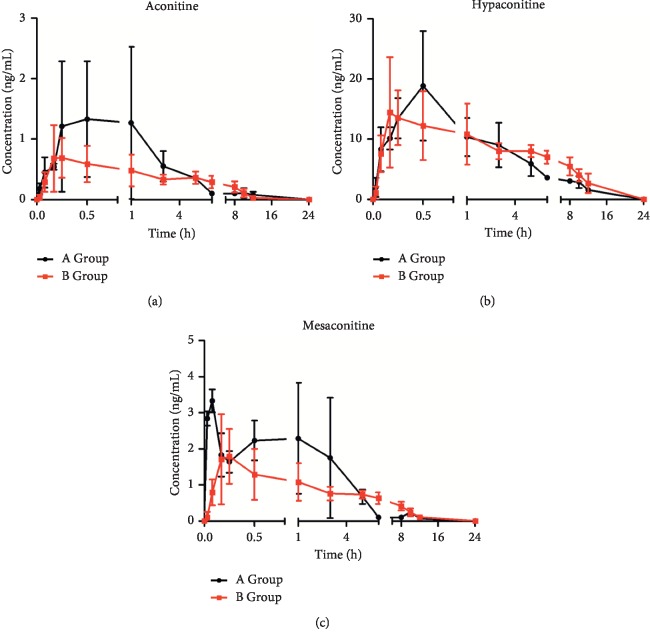
Mean plasma concentration-time curves of aconitine, mesaconitine, and hypaconitine in different groups (mean ± SD, *n* = 6). Group A received *Aconiti Lateralis Radix Praeparata* extract through oral administration. Group B received *Aconiti Lateralis Radix Praeparata* extract and red ginseng extract through oral administration. (a) Aconitine. (b) Hypaconitine. (c) Mesaconitine.

The toxicity of *Aconiti Lateralis Radix Praeparata* is mainly attributed to its diester diterpenoid aconitines such as aconitine, hypaconitine, and mesaconitine [23, 24]. According to reports, diester diterpenoid aconitines of *Aconiti Lateralis Radix Praeparata* are substrates of CYP3A4 metabolic enzymes [10]. Further pharmacokinetic studies were carried out on *Aconiti Lateralis Radix Praeparata* and its combination with red ginseng (Figure 3). C_max_ and AUC_(0-∞)_ of aconitine, hypaconitine, and mesaconitine of *Aconiti Lateralis Radix Praeparata*-red ginseng were decreased as compared to those of *Aconiti Lateralis Radix Praeparata* administration. Therefore, red ginseng may accelerate the metabolism of toxic components in *Aconiti Lateralis Radix Praeparata* by inducing the activity of CYP3A4 enzymes. This experiment provides a theoretical basis for explaining the interaction mechanism of *Aconiti Lateralis Radix Praeparata* combined with red ginseng, which would provide reference for rational usage of red ginseng and *Aconiti Lateralis Radix Praeparata* in clinic.

## 4. Conclusion

In this study, a sensitive and rapid LC-MS/MS method was developed and validated for the simultaneous determination of eight probe drugs in rat plasma to evaluate the effect of *Aconiti Lateralis Radix Praeparata* and its combination with red ginseng on rat CYP450 enzymes. The results indicated that *Aconiti Lateralis Radix Praeparata* may inhibit the activity of CYP2A6, CYP2C19, CYP2B6, CYP1A2, CYP3A4, and CYP2C9 in rats. Comparison between the two groups of *Aconiti Lateralis Radix Praeparata* with or without red ginseng showed that red ginseng may inhibit the activity of CYP2D6 and CYP2B6 while inducing the activity of CYP1A2, CYP3A4, and CYP2C9 enzymes.

## Figures and Tables

**Table 1:** MS parameters of the eight probe drugs and IS.

Probe drug	Precursor ion (*m*/*z*)	Product ion (*m*/*z*)	Frag. (V)	C.E. (V)
Omeprazole	346.2	136.1	75	35
Dapsone	249.1	92.1	125	21
Dextromethorphan	272.2	147.1	115	30
Phenacetin	180.1	110.1	105	20
Tolbutamide	271.1	91.0	80	30
Bupropion	240.1	184.1	85	7
Amodiaquine	356.2	283.1	96	15
7-Hydroxycoumarin	163.0	107.1	95	20
Diazepam (IS)	285.0	193.0	105	30

**Table 2:** The calibration curves and LLOQs of the eight probe drugs.

Probe drug	Regression equation	Correlation coefficients (*r*)	Linear range (ng/mL)	LLOQ (ng/mL)
7-Hydroxycoumarin	*y* = 0.185218*x* + 0.003722	0.9964	10–5000	2
Omeprazole	*y* = 0.952998*x* + 0.015885	0.9920	4–2000	1
Dextromethorphan	*y* = 0.888828*x* + 0.008993	0.9919	1–500	1
Bupropion	*y* = 1.871081*x* + 0.001977	0.9910	2–1000	1
Phenacetin	*y* = 1.442258*x* + 0.009962	0.9919	2–1000	1
Dapsone	*y* = 0.376316*x* + 0.001369	0.9961	1–500	1
Tolbutamide	*y* = 0.499142*x* + 0.004604	0.9984	6–3000	1
Amodiaquine	*y* = 1.707230*x* + 0.230893	0.9937	2–1000	1

**Table 3:** Precision and accuracy of the eight probe drugs in rat plasma (*n* = 6).

Probe drug	Spiked conc. (ng/mL)	Intraday			Interday		
		Measured conc. (ng/mL)	Precision (RSD, %)	Accuracy (RE, %)	Measured conc. (ng/mL)	Precision (RSD, %)	Accuracy (RE, %)
7-Hydroxycoumarin	25	22.0 ± 2.0	9.1	−12.0	24.6 ± 2.5	10.2	−1.6
	500	523.1 ± 33.4	6.4	4.6	517.0 ± 26.6	5.1	3.4
	5000	4344.9 ± 251.1	5.8	−13.1	4375.8 ± 218.6	5.0	−12.5

Omeprazole	10	10.0 ± 0.8	8.0	0.0	10.6 ± 0.8	7.5	6.0
	200	228.7 ± 8.9	3.9	14.4	222.6 ± 10.5	4.7	11.3
	2000	1720.9 ± 96.0	5.6	−14.0	1772.9 ± 104.2	6.0	−11.4

Dextromethorphan	2.5	2.3 ± 0.3	13.0	−8.0	2.5 ± 0.3	12.0	0.0
	50	52.7 ± 2.8	5.3	5.4	50.8 ± 2.6	5.1	1.6
	500	476.8 ± 41.3	8.7	−4.6	489.1 ± 31.1	6.4	−2.2

Bupropion	5	4.3 ± 0.2	4.7	−14.0	4.8 ± 0.2	4.2	−4.0
	100	98.6 ± 7.7	7.8	−1.4	107.7 ± 11.0	10.2	7.7
	1000	1063.5 ± 80.3	7.6	6.4	1052.9 ± 101.3	9.6	5.3

Phenacetin	5	4.5 ± 0.5	11.1	−10.0	5.0 ± 0.6	12.0	0.0
	100	98.3 ± 6.2	6.3	−1.7	101.1 ± 7.8	7.7	1.1
	1000	923.1 ± 49.8	5.4	−7.7	913.0 ± 53.6	5.9	−8.7

Dapsone	2.5	2.3 ± 0.1	4.3	−8.0	2.6 ± 0.3	11.5	4.0
	50	55.4 ± 2.9	5.2	10.8	52.9 ± 3.9	7.4	5.8
	500	492.7 ± 36.8	7.5	−1.5	482.6 ± 31.8	6.6	−2.5

Tolbutamide	15	14.5 ± 0.6	4.1	−3.3	15.4 ± 0.6	3.9	2.7
	300	317.6 ± 12.9	4.1	5.9	311.0 ± 12.8	4.1	3.7
	3000	3034.3 ± 178.8	5.9	1.1	3029.8 ± 140.4	4.6	1.0

Amodiaquine	5	5.5 ± 0.3	5.5	10.0	4.9 ± 0.3	6.1	−2
	100	113.1 ± 3.7	3.3	13.1	112.1 ± 4.7	4.2	12.1
	1000	860.4 ± 41.8	4.9	−14.0	913.2 ± 60.9	6.7	−8.7

**Table 4:** Extraction recoveries and matrix effects of the eight probe drugs in rat plasma (*n* = 6).

Probe drug	Spiked conc. (ng/mL)	Extraction recovery (%)	RSD (%)	Matrix effect (%)	RSD (%)
7-Hydroxycoumarin	25	102.6 ± 4.6	4.5	81.5 ± 1.7	2.1
	500	106.4 ± 7.8	7.3	93.6 ± 5.1	5.4
	5000	109.4 ± 5.0	4.6	95.4 ± 1.0	1.0

Omeprazole	10	90.1 ± 6.0	6.7	95.9 ± 6.4	6.7
	200	103.1 ± 6.6	6.4	96.3 ± 4.4	4.6
	2000	99.6 ± 2.7	2.7	99.4 ± 1.2	1.2

Dextromethorphan	2.5	109.3 ± 9.4	8.6	105.6 ± 6.7	6.3
	50	106.9 ± 4.5	4.2	94.7 ± 2.5	2.6
	500	99.3 ± 3.1	3.1	100.4 ± 1.8	1.8

Bupropion	5	85.0 ± 9.9	11.6	93.3 ± 7.1	7.6
	100	85.2 ± 3.1	3.6	90.6 ± 1.3	1.4
	1000	87.8 ± 7.8	8.9	100.0 ± 0.8	0.8

Phenacetin	5	94.7 ± 8.3	8.8	92.9 ± 4.6	5.0
	100	106.4 ± 9.1	8.6	92.5 ± 5.0	5.4
	1000	106.9 ± 5.9	5.5	96.4 ± 1.5	1.6

Dapsone	2.5	98.7 ± 8.5	8.6	90.3 ± 5.7	6.3
	50	103.4 ± 4.9	4.7	89.3 ± 1.8	2.0
	500	103.9 ± 3.8	3.7	97.7 ± 1.6	1.6

Tolbutamide	15	86.5 ± 6.5	7.5	87.3 ± 1.6	1.8
	300	85.9 ± 3.2	3.7	90.2 ± 2.5	2.8
	3000	91.9 ± 2.7	2.9	93.9 ± 0.8	0.9

Amodiaquine	5	91.5 ± 4.5	4.9	85.5 ± 4.9	5.7
	100	93.8 ± 9.2	9.8	100.0 ± 9.9	9.9
	1000	95.4 ± 3.9	4.1	102.2 ± 3.1	3.0

**Table 5:** Stability results of the eight probe drugs in rat plasma (*n* = 6).

Probe drug	Spiked conc. (ng/mL)	4 h at room temperature		3 freeze-thaw cycles		12 h in autosampler		−70°C for 14 days	
		Measured conc. (ng/mL)	RSD (%)	Measured conc. (ng/mL)	RSD (%)	Measured conc. (ng/mL)	RSD (%)	Measured conc. (ng/mL)	RSD (%)
7-Hydroxycoumarin	25	25.4 ± 1.2	4.7	24.6 ± 0.6	2.4	27.1 ± 2.0	7.4	25.7 ± 1.4	5.4
	500	558.8 ± 11.9	2.1	498.5 ± 33.8	6.8	529.9 ± 7.2	1.4	537.4 ± 27.5	5.1
	5000	5063.6 ± 247.0	4.9	4263.2 ± 145.5	3.4	4472.6 ± 505.3	11.3	4358.0 ± 117.4	2.7

Omeprazole	10	11.1 ± 0.5	4.5	11.2 ± 0.3	2.7	10.5 ± 1.0	9.5	11.2 ± 0.1	0.9
	200	228.8 ± 4.5	2.0	228.9 ± 9.1	4.0	223.6 ± 8.3	3.7	228.8 ± 2.6	1.1
	2000	2061.6 ± 77.2	3.7	1730.0 ± 57.4	3.3	1842.8 ± 100.4	5.4	1844.5 ± 24.5	1.3

Dextromethorphan	2.5	2.5 ± 0.2	8.0	2.2 ± 0.1	4.5	2.6 ± 0.3	9.8	2.1 ± 0.1	4.8
	50	55.9 ± 0.8	1.4	51.3 ± 4.0	7.8	52.2 ± 3.7	7.2	56.0 ± 1.4	2.5
	500	560.0 ± 18.9	3.4	469.8 ± 17.5	3.7	503.9 ± 51.6	10.2	493.9 ± 14.4	2.9

Bupropion	5	4.9 ± 0.6	12.2	4.4 ± 0.5	11.4	4.2 ± 0.2	4.5	5.4 ± 0.2	3.7
	100	114.0 ± 3.5	3.1	112.0 ± 10.7	9.6	98.3 ± 12.6	12.9	104.1 ± 13.9	13.4
	1000	1135.8 ± 50.7	4.5	1008.7 ± 144.1	14.3	959.1 ± 44.4	4.6	1128.5 ± 73.4	6.5

Phenacetin	5	5.1 ± 0.2	3.9	4.4 ± 0.3	6.8	5.0 ± 0.3	5.5	4.9 ± 0.4	8.2
	100	113.1 ± 3.1	2.7	102.3 ± 9.8	9.6	100.1 ± 4.7	4.7	111.2 ± 5.7	5.1
	1000	1021.4 ± 49.1	4.8	867.7 ± 31.3	3.6	850.6 ± 35.0	4.1	873.7 ± 32.2	3.7

Dapsone	2.5	2.5 ± 0.2	8.0	2.6 ± 0.2	7.7	2.6 ± 0.4	13.5	2.5 ± 0.2	8.0
	50	56.0 ± 1.8	3.2	55.7 ± 3.7	6.6	52.1 ± 2.4	4.5	56.1 ± 1.4	2.5
	500	559.7 ± 33.1	5.9	487.6 ± 15.8	3.2	470.8 ± 64.1	13.6	528.2 ± 21.0	4.0

Tolbutamide	15	14.7 ± 0.7	4.8	13.0 ± 0.5	3.8	15.3 ± 0.2	1.6	14.8 ± 0.3	2.0
	300	332.9 ± 10.6	3.2	292.4 ± 19.4	6.6	318.6 ± 16.9	5.3	316.2 ± 10.9	3.4
	3000	3397.6 ± 217.0	6.4	2725.3 ± 114.1	4.2	3425.5 ± 170.8	5.0	2864.3 ± 40.3	1.4

Amodiaquine	5	4.5 ± 0.4	8.9	5.0 ± 0.7	14.0	5.4 ± 0.3	5.1	5.4 ± 0.2	3.7
	100	112.1 ± 5.1	4.5	91.8 ± 5.4	5.9	106.3 ± 2.0	1.9	86.6 ± 6.8	7.9
	1000	926.3 ± 3.6	0.4	861.1 ± 38.7	4.5	917.9 ± 53.7	5.9	851.3 ± 6.6	0.8

**Table 6:** Pharmacokinetic parameters of the eight probe drugs in different groups (*n* = 6).

Probe drug	Group	C_max_ (ng/mL)	*t*_1/2_ (h)	AUC_(0-24)_ (h·ng/mL)	AUC_(0-∞)_ (h·ng/mL)	MRT_(0-24)_ (h)	MRT_(0-∞)_ (h)
7-Hydroxycoumarin	Group A	3310.91 ± 348.65	0.03 ± 0.01	212.41 ± 38.95	214.88 ± 39.27	0.06 ± 0.02	0.09 ± 0.06
	Group B	5360.83 ± 701.87^∗∗^	0.03 ± 0.01	387.45 ± 68.22^∗∗^	394.89 ± 68.81^∗∗^	0.10 ± 0.06	0.17 ± 0.14
	Group C	4932.90 ± 675.99	0.03 ± 0.01	355.16 ± 84.91	361.22 ± 83.06	0.08 ± 0.02	0.17 ± 0.22

Omeprazole	Group A	987.61 ± 72.73	0.15 ± 0.03	252.70 ± 43.54	256.31 ± 43.28	0.32 ± 0.07	0.36 ± 0.11
	Group B	1286.26 ± 151.01^∗∗^	0.02 ± 0.02^∗∗^	372.49 ± 71.09^∗∗^	386.43 ± 72.30^∗∗^	0.46 ± 0.16	0.59 ± 0.27
	Group C	1574.58 ± 170.41^#^	0.02 ± 0.01	337.91 ± 62.29	342.83 ± 60.77	0.31 ± 0.05	0.34 ± 0.04

Dextromethorphan	Group A	172.16 ± 13.69	0.42 ± 0.09	156.08 ± 26.13	161.46 ± 29.28	1.51 ± 0.26	1.74 ± 0.33
	Group B	165.50 ± 15.41	0.41 ± 0.03	171.04 ± 31.51	176.99 ± 35.12	2.06 ± 0.76	2.58 ± 1.49
	Group C	248.50 ± 19.2 9^##^	0.03 ± 0.02^##^	290.03 ± 38.27^##^	340.32 ± 92.03^##^	3.35 ± 0.89^#^	10.28 ± 11.90

Bupropion	Group A	138.32 ± 28.61	0.22 ± 0.11	67.70 ± 11.50	69.72 ± 11.44	1.01 ± 0.16	1.20 ± 0.23
	Group B	149.44 ± 29.54	0.25 ± 0.13	85.71 ± 14.43^∗^	88.31 ± 14.45^∗^	1.09 ± 0.18	1.29 ± 0.23
	Group C	384.52 ± 73.86^##^	0.11 ± 0.05^#^	139.45 ± 28.21^##^	141.53 ± 28.29^##^	0.94 ± 0.06	1.03 ± 0.08^#^

Phenacetin	Group A	495.83 ± 36.15	0.13 ± 0.02	116.03 ± 17.62	118.18 ± 17.65	0.28 ± 0.06	0.30 ± 0.08
	Group B	743.88 ± 71.09^∗∗^	0.13 ± 0.03	187.04 ± 35.84^∗∗^	190.07 ± 34.61^∗∗^	0.34 ± 0.03^∗^	0.38 ± 0.08
	Group C	689.54 ± 59.76	0.03 ± 0.02^##^	141.09 ± 27.32^#^	143.90 ± 27.08^#^	0.31 ± 0.05	0.34 ± 0.04

Dapsone	Group A	458.48 ± 60.11	0.04 ± 0.03	1347.37 ± 179.94	1369.79 ± 184.98	4.73 ± 0.47	5.14 ± 0.61
	Group B	535.58 ± 42.55^∗^	0.27 ± 0.27	2400.17 ± 561.81^∗∗^	2497.74 ± 588.53^∗∗^	6.01 ± 0.78^∗∗^	7.02 ± 1.43^∗^
	Group C	565.45 ± 40.30	0.03 ± 0.02	1291.00 ± 254.39^##^	1312.41 ± 272.61^##^	4.70 ± 0.66^#^	5.07 ± 0.97^#^

Tolbutamide	Group A	2144.68 ± 97.44	0.09 ± 0.06	15770.98 ± 1759.31	17824.22 ± 2236.80	7.72 ± 0.31	10.91 ± 1.03
	Group B	3457.97 ± 177.28^∗∗^	0.10 ± 0.08	21745.37 ± 3425.25^∗∗^	32421.98 ± 9258.81^∗^	8.85 ± 0.64^∗∗^	21.88 ± 13.86
	Group C	2616.79 ± 105.40^##^	0.06 ± 0.02	11848.93 ± 1187.25^##^	12767.39 ± 1304.87^##^	7.02 ± 0.22^##^	8.92 ± 0.47^#^

Amodiaquine	Group A	143.49 ± 12.30	0.04 ± 0.03	177.26 ± 50.00	196.00 ± 57.29	5.69 ± 1.12	8.23 ± 2.14
	Group B	127.07 ± 19.29	0.07 ± 0.03	138.39 ± 60.47	157.45 ± 60.55	4.16 ± 2.28	6.23 ± 3.19
	Group C	216.91 ± 43.19^##^	0.02 ± 0.03^#^	189.37 ± 18.90	235.24 ± 71.91	5.84 ± 0.83	12.49 ± 8.33

^*∗*^ *P* < 0.05, B different from group A; ^*∗∗*^*P* < 0.01, B significantly different from group A. ^#^*P* < 0.05, C different from group B;^##^*P* < 0.01, C significantly different from group B.

## Data Availability

The data used to support the findings of this study are available from the corresponding author upon request.
